# Sequence Fingerprints of MicroRNA Conservation

**DOI:** 10.1371/journal.pone.0048256

**Published:** 2012-10-24

**Authors:** Bing Shi, Wei Gao, Juan Wang

**Affiliations:** 1 Department of Cardiology, Peking University Third Hospital, Beijing, China; 2 Department of Biomedical Informatics, Peking University Health Science Center, Beijing, China; The John Curtin School of Medical Research, Australia

## Abstract

It is known that the conservation of protein-coding genes is associated with their sequences both various species, such as animals and plants. However, the association between microRNA (miRNA) conservation and their sequences in various species remains unexplored. Here we report the association of miRNA conservation with its sequence features, such as base content and cleavage sites, suggesting that miRNA sequences contain the fingerprints for miRNA conservation. More interestingly, different species show different and even opposite patterns between miRNA conservation and sequence features. For example, mammalian miRNAs show a positive/negative correlation between conservation and AU/GC content, whereas plant miRNAs show a negative/positive correlation between conservation and AU/GC content. Further analysis puts forward the hypothesis that the introns of protein-coding genes may be a main driving force for the origin and evolution of mammalian miRNAs. At the 5′ end, conserved miRNAs have a preference for base U, while less-conserved miRNAs have a preference for a non-U base in mammals. This difference does not exist in insects and plants, in which both conserved miRNAs and less-conserved miRNAs have a preference for base U at the 5′ end. We further revealed that the non-U preference at the 5′ end of less-conserved mammalian miRNAs is associated with miRNA function diversity, which may have evolved from the pressure of a highly sophisticated environmental stimulus the mammals encountered during evolution. These results indicated that miRNA sequences contain the fingerprints for conservation, and these fingerprints vary according to species. More importantly, the results suggest that although species share common mechanisms by which miRNAs originate and evolve, mammals may develop a novel mechanism for miRNA origin and evolution. In addition, the fingerprint found in this study can be predictor of miRNA conservation, and the findings are helpful in achieving a clearer understanding of miRNA function and evolution.

## Introduction

Gene evolution has been well investigated, and the conservation of genes has been found to be associated with many biological factors [1]. microRNAs (miRNAs) are small non-coding regulatory RNAs that have been identified in recent years [2]. They have important biological functions and show strong association with various diseases [3]. Similar with protein-coding genes, studies on miRNA conservation are important in understanding not only their function and genomic organization but also their relation to human disease and medicine [4]. Indeed, many factors have been revealed to be correlated with miRNA conservation in recent years. For example, many miRNAs tend to be conserved during evolution [2], [5], [6]. Zhang et al. reported that human-specific miRNAs tend to evolve rapidly [4]. miRNA precursor stem loops show significantly increased mutational robustness [7]. The rapid evolution of an X-linked microRNA cluster [8] and the Alu-mediated rapid expansion of miRNA genes [9] in primates have been observed. Vazquez et al. found that recently evolved miRNAs tend to be longer than ancient miRNAs in Arabidopsis [10]. Szollosi et al. suggested that the conservation of miRNAs may be associated with selection for environmental robustness [11]. Piriyapongsa et al. revealed that transposable element (TE)-derived miRNAs are less conserved than non-TE-derived miRNAs in human [12]. Liang et al. observed that highly expressed human microRNA genes tend to be conserved [13]. Recently, de Wit et al. discovered hairpin shifting, a novel mode of miRNA evolution [14].

The above studies have uncovered several important evolutionary insights. However, the relationship between some important sequence features and miRNA conservation has not been investigated to date. Many questions about the relationships of miRNA conservation and their sequence features remain unknown. Is the base content of miRNAs correlated with their conservation? Do the sequence features of mature miRNA (MIR) sequences and miRNA precursors (pre-miR) have similar correlation with miRNA conservation? Do conserved miRNAs tend to have a high GC content? Are there any differences among species? Does the cleavage process of MIRs have an affect on their conservation?

To address these questions, we analyzed the correlation between miRNA conservation and various miRNA sequence features, including ACGU content of MIRs, pre-miRs, and non-MIR sequences, and the base content at cleavage sites. The results show that these sequence features are significantly correlated with miRNA conservation, and different species unexpectedly show different and even opposite correlation patterns. Our analysis further revealed that the introns of protein-coding genes and the need of functional diversity of might be driving forces for miRNA origin and evolution in mammals.

## Results

### Species show specific patterns of correlation between miRNA conservation and their base content

**Figure 1:**
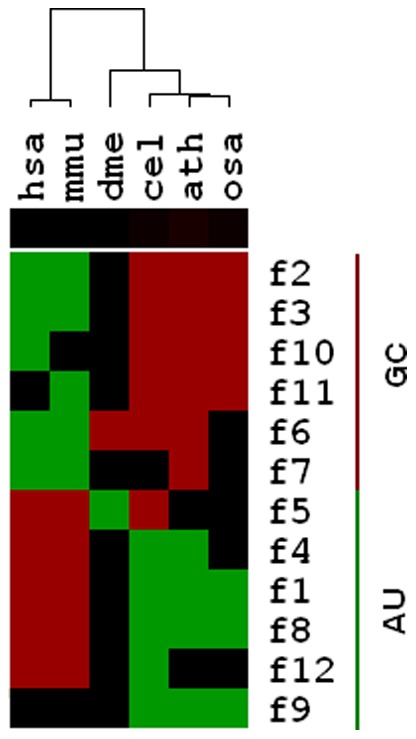
Clustering heatmap of the correlation patterns between base content and miRNA conservation in six species. A red element indicates a positive correlation between the corresponding sequence features and miRNA conservation for a given species. In contrast, a green element indicates a negative correlation, and a black one represents a non-significant correlation. Features F1–F4 are the base contents of A, C, G, and U of the miRNA precursors. Features F5–F8 are the base contents of A, C, G, and U of the mature miRNAs. Features F9–F12 are the base contents of A, C, G, and U of non-mature miRNAs.

We first performed correlation analysis for miRNA conservation and their base content (Features 1–12). The results showed that miRNA conservation is significantly correlated with the sequence features in the six species (Table 1). For human and mouse, miRNA conservation showed a significantly positive correlation with AU content but a significantly negative correlation with GC content in all types of sequences (MIR, pre-miR, and non-MIR). The above correlations disappeared in fly but began to be reversed from worm (Table 1, Figure 1). Compared with two mammals, in two plants, Arabidopsis and rice, the correlation patterns between miRNA conservation and base content are nearly totally reversed (Table 1). For protein-coding genes, however, conserved genes tend to have an increased GC content in mammals, birds [15], yeast, Arabidopsis [16], and rice [17]. Obviously, this situation in mammal miRNAs is different from that in worm and plant miRNAs and that in protein-coding genes. This suggested that the forces connecting base content and evolution in miRNAs might be diverged in miRNAs, especially in mammal miRNAs.

**Table 1:** Correlation coefficients and significant values (P values) of miRNA conservation and sequence features (F1-F12) in six species. Features F1–F4 are the base contents of A, C, G, and U of miRNA precursors.

	Human	Mouse	Fly	Worm	Arabidopsis	Rice
F1	0.14,5.7e-4	0.15,4.0e-4	0.004,0.96	−0.27,1.4e-3	−0.46,6.4e-11	−0.24,4.8e-6
F2	−0.25,6.1e-10	−0.19,8.3e-06	−0.01,0.94	0.32,1.1e-4	0.35,1.4e-06	0.13,0.02
F3	−0.13,1.8e-3	−0.22,5.3e-07	−0.01,0.90	0.21,0.01	0.35,1.1e-06	0.20,1.4e-4
F4	0.26,3.4e-11	0.24,2.7e-08	0.02,0.78	−0.20,0.02	−0.12,0.11	−0.01,0.88
F5	0.23,3.6e-09	0.17,1.1e-4	−0.19,0.03	0.12,0.16	−0.01,0.85	−0.02,0.69
F6	−0.15,2.4e-4	−0.18,4.5e-05	0.10,0.24	0.14,0.10	0.21,3.8e-3	0.07,0.20
F7	−0.16,5.7e-05	−0.18,1.8e-05	−0.005,0.96	0.002,0.97	0.33,4.5e-06	0.08,0.11
F8	0.18,9.9e-06	0.27,2.0e-10	−0.01,0.89	−0.21,0.01	−0.46,4.9e-11	−0.13,0.02
F9	0.07,0.08	0.07,0.08	0.09,0.32	−0.30,2.8e-4	−0.43,8.5e-10	−0.24,3.3e-06
F10	−0.19,1.3e-06	−0.09,0.03	−0.09,0.32	0.28,7.3e-4	0.25,6.9e-4	0.12,0.03
F11	−0.05,0.22	−0.12,7.7e-3	−0.04,0.67	0.21,0.01	0.19,8.7e-3	0.17,8.3e-4
F12	0.23,4.9e-09	0.15,5.6e-4	0.04,0.65	−0.12,0.17	0.06,0.44	0.02,0.68

Features F5–F8 are the base contents of A, C, G, and U of mature miRNAs. Features F9–F12 are the base contents of A, C, G, and U of non-mature sequences. Correlation analysis was performed for each miRNA feature and miRNA conservation for each species. The numbers in each cell mean the correlation coefficient and p value of the correlation analysis.

### Species show specific patterns of correlation between miRNA conservation and the bases at cleavage sites

**Figure 2:**
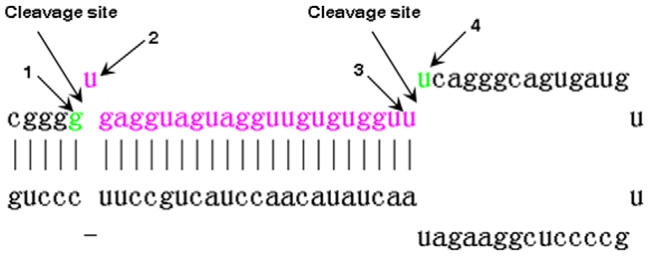
A diagram of bases at miRNA cleavage sites. The colored sequence represents the mature miRNA. The base pointed by “2” is the second base at cleavage sites and is also the base of the 5′ end.

**Figure 3:**
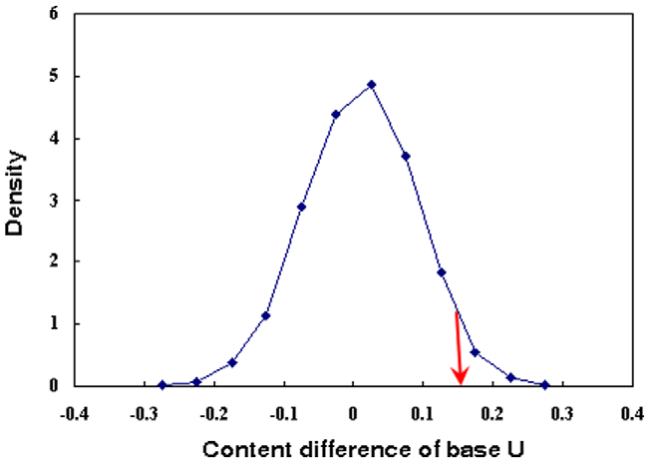
A diagram indicating the significance of different base preferences at the 5′ end for conserved miRNAs and less-conserved miRNAs in human. The blue curve is the random distribution of the difference of the base U fraction between conserved miRNAs and less-conserved miRNAs. The red arrow represents the real difference of the base U fraction between conserved miRNAs and less-conserved miRNAs.

The location of caspase cleavage sites has been reported to often have specific structural characteristics [18]. This gives us clues that the cleavage sites of miRNAs may also have specific features for miRNA conservation. To investigate this, we first classified miRNAs into two groups: miRNAs that have at least one other homolog (conserved miRNAs) and miRNAs that do not have any homolog (termed as less-conserved miRNAs). We next analyzed the distribution of ACGU in the four bases at the cleavage sites (Figure 2). The result shows that conserved miRNAs have a greater fraction of U at the 5′ terminus than less-conserved miRNAs in mammals (Figure S1). For example, 45.5% of conserved miRNAs have a base U at the 5′ terminus in human, whereas only 30.2% of less-conserved miRNAs have a base U at the 5′ terminus. This difference was further confirmed to be significant (P = 0.02, randomization test; Figure 3). Moreover, miRNAs with a base U at the 5′ terminus are more conserved than miRNAs with a non-U base at the 5′ terminus (P = 2.0×10^−4^, Wilcoxon test). As a comparison, the miRNAs of insects and plants do not have the above pattern.

### Hypothesis on the intron-driven origin of miRNAs in mammals

**Figure 4:**
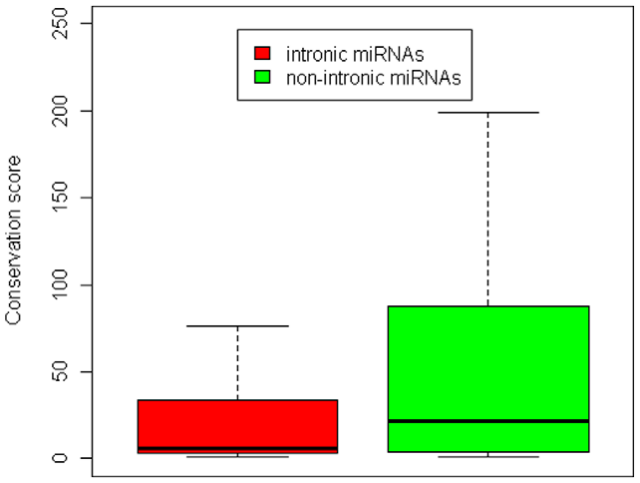
A comparison of the miRNA conservation score between intronic miRNAs and non-intronic miRNAs.

The investigation of why animals and plants show opposite patterns in the correlation of base content and conservation of miRNAs is interesting. As described above, unlike protein-coding genes in both animals and plants and unlike miRNAs in plants which show positive correlation between GC content and their conservation, the miRNAs in mammals show a negative correlation between GC content and their conservation, an opposite pattern with protein and plant miRNAs. How does this occur? A big difference in the genome location of miRNAs in animals and plants may give us the clues. Most plant miRNAs are located in intergenic regions, whereas many animal miRNAs are located in the introns of protein-coding genes. Considering that protein-coding genes have a higher fraction of GC content than other DNA regions, as introduced above, novel miRNAs will have a higher fraction of GC content than conserved miRNAs in animals if less-conserved miRNAs have a higher probability of intron-driven origins. We first confirmed this in human. We calculated the fractions of intronic miRNAs in conserved miRNAs and less-conserved miRNAs, respectively. Indeed, less-conserved miRNAs have a higher fraction (46.5%) of intron origin than conserved miRNAs (38.2%). We further compared the conservation scores of intronic miRNAs and non-intronic miRNAs. As expected, intronic miRNAs show significantly less conservation than non-intronic miRNAs (P = 1.3×10^−9^, Wilcoxon test; Figure 4). This result suggested that the higher enrichment of GC in novel miRNAs may mainly result from their enrichment in introns. Therefore, investigating the correlation between base content and miRNA conservation in the context of introns and non-introns, respectively, will be interesting. As a result, non-intronic miRNAs show consistent patterns with plant miRNAs. For example, the content of base A shows a significantly negative correlation with miRNA conservation (R = −0.33, P = 7.0×10^−11^ for miRNA precursors; R = −0.15, P = 0.004 for non-mature sequences, which are the rest sequences of miRNA precursors after removing the mature ones). For intronic miRNAs, however, they still tend to show opposite patterns compared with non-intronic miRNAs, miRNAs in plants, and protein-coding genes. For example, base C is negatively correlated with miRNA conservation (R = −0.17, P = 0.009 for miRNA precursors; R = −27, P =  = 2.7×10^−5^ for mature miRNAs). This result suggested that intronic miRNAs prefer to originate from younger protein-coding genes rather than older ones. We repeated the above analysis in mouse, and the main results are the same (data not shown). All these findings suggested the hypothesis that the introns of protein-coding genes, especially young protein-coding genes, may be a driving force for miRNA origin.

### Hypothesis on functional diversity-driven miRNA evolution in mammals

**Figure 5:**
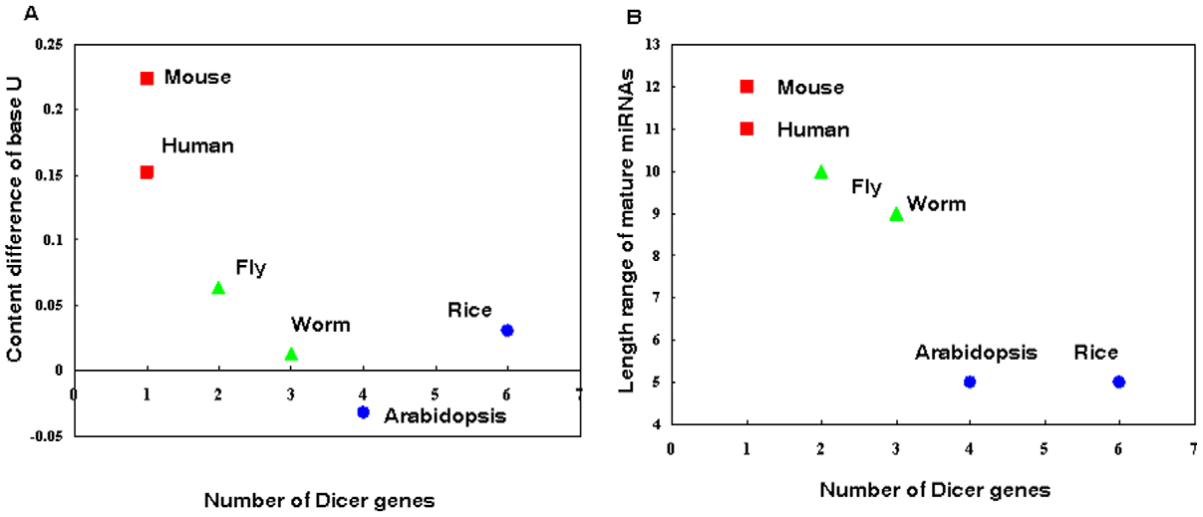
Correlations between the number of Dicer genes a species has and the miRNA sequence features among the six species, including the content difference of the base U fraction at the 5′ end for conserved miRNAs and less-conserved miRNAs (A) and length range of mature miRNAs (B).

**Figure 6:**
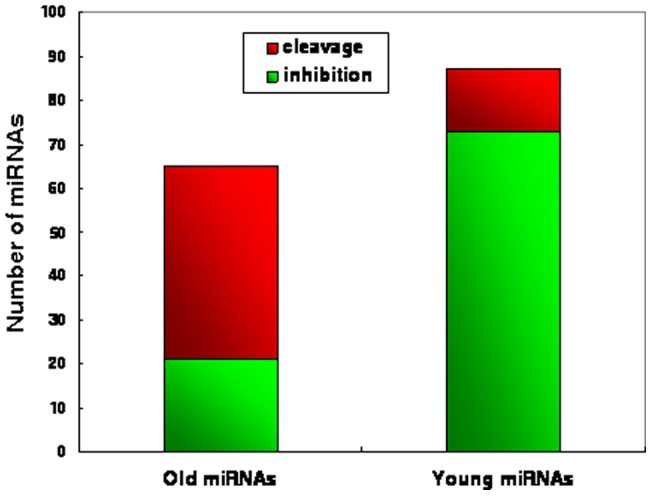
A comparison of the silencing mechanism distribution in old miRNAs (highly conserved miRNAs) and young miRNAs (lowly conserved miRNAs).

We have shown that in mammals, conserved miRNAs have a preference for base U at the 5′ end, whereas this pattern does not exist in insects and plants. However, what are the reasons for this bias? Why do evolutionary new miRNAs use base U less at the 5′ terminus in mammals? Studies have suggested that gene expression by miRNAs can explain differences in organism complexity [19], [20]. During evolution, highly sophisticated organisms need to develop more diverse biological functions in order to meet the challenges of more complex tasks. The change of the base fraction at the 5′ terminus may be driven by the need for miRNA functional diversity. The evolution of Dicer genes, critical genes in small RNA biogenesis, may provide some clues. Different species have different numbers of Dicer genes. For example, the six species used in this study have one (human and mouse), two (fly), three (worm), four (Arabidopsis), and six (rice) Dicer genes, respectively. This suggested that during evolution, the number of Dicer genes tends to decrease and that the functions of multiple Dicer genes are integrated into one Dicer gene in mammals. Therefore, the mammal Dicer gene tends to have greater functional diversity, which may result in greater diversity in miRNA biogenesis and function. We first confirmed this by investigating the change of the base U fraction at the 5′ end, the most critical cut point of Dicer. From plants to mammals, the differences of the base U fraction between conserved and less-conserved miRNAs increase as the number of Dicer genes decreases (R = −0.81, P = 0.05, Spearman's correlation; Figure 5A). Moreover, mammals also showed greater diversity in miRNA length (R = −0.97, P = 0.001, Spearman's correlation; Figure 5B). In miRNA sorting, Ago1 has been shown to have a strong preference for base U at the 5′ end, whereas Ago2 and Ago4 have a preference for other bases at the 5′ end [21]. The changes in base content at the 5′ end may trigger a switch in miRNA silencing mechanisms, from cleavage to translation inhibition [21]. miRNA is known to silence targets by two mechanisms, cleavage and translation inhibition. The target cleavage often results from perfect miRNA-mRNA hybrids, whereas translation inhibition normally results from imperfect miRNA-mRNA hybrids. The binding style means that miRNAs using the translation inhibition mechanism normally have bigger target numbers. Therefore, they have greater functional diversity than miRNAs using the cleavage mechanism. Indeed, reports have indicated that typically, animal miRNAs regulate targets by translation inhibition so they normally have dozens of targets. Plant miRNAs, in contrast, typically regulate targets by cleavage so they normally have a limited number of targets [21]. This supported the hypothesis that the origin of miRNAs in highly sophisticated organisms (i.e., human) might be driven by the increasing need for functional diversity. As a result, young miRNAs tend to use the non-U base at 5′ ends, which then triggers the switch in miRNA regulation mechanisms and alters functional diversity. To confirm this, we investigated the relationship between miRNA regulation mechanisms and miRNA conservation in human using a number of experimentally supported miRNA regulation mechanism data. In particular, we obtained data on experimentally supported miRNA regulation mechanisms from TarBase [22], then we classified these miRNAs into two groups: the highly conserved group (old miRNAs) and the lowly conserved group (young miRNAs). As expected, young miRNAs have a strong preference for the mechanism of translation inhibition compared with old miRNAs (translation inhibition fraction in two groups: 83.9% vs. 32.3%, P = 7.0×10^−11^, Fisher's exact test; Figure 6). This suggested that young miRNAs in mammals tend to have diverse functions, supporting the hypothesis that the pressure of functional diversity during the evolution of highly sophisticated organisms might be a force influencing miRNA origin.

### Network context of miRNAs have effects on their evolution

Previous studies have shown that molecules evolution is affected by the biological networks of these molecules [23], [24]. For miRNAs, they also show preference when targeting genes in the context of biological network, for example signaling network [25]. Therefore, it will be interesting whether miRNAs show different patterns of evolution in the context of biological network. For doing so, we obtained the predicted miRNA target from TargetScan. We then mapped miRNAs into a human signaling network [26] through miRNA targets. We further identified top 10% miRNAs that predominantly target extracellular molecules, membrane molecules, cytosol molecules, and nucleus molecules, respectively. As a result, miRNAs that regulate different network context targets show significantly different evolutionary patterns. miRNAs regulating extracellular molecules are the most conserved ones, followed by miRNAs regulating membrane molecules, cytosol molecules, and nucleus molecules. From extracellular space to nucleus, these miRNAs show decreasing conservation. For example, from extracellular space to nucleus, the four groups of miRNAs have 14, 12, 6, and 2 most conserved miRNAs, respectively.

## Discussion

In summary, we have revealed that miRNA sequence features contain information on miRNA conservation, suggesting that these features might be the fingerprint for miRNA evolution. More interestingly, different species show different and even opposite patterns of fingerprints, indicating that although common mechanisms of evolution exist in these species, novel evolutionary rules occur in some of the species during evolution.

Analysis of the correlation between base content and miRNA conservation revealed that mammals and plants show opposite patterns. Furthermore, evidence from intronic miRNAs and their base content distribution put forward the hypothesis on intron-driven miRNA origin in mammals. The reasons why many mammal miRNAs originate from the introns of protein-coding genes, especially evolutionary young protein-coding genes, remain largely unknown. This origin of miRNAs is assumed to possess some advantages. For example, intronic miRNAs with their host genes may form transcription units and co-play roles in common tasks.

Analysis of the bases at miRNA cleavage sites showed that conserved miRNAs have a preference for base U at the 5′ end in mammals, whereas less-conserved miRNAs have a preference for non-U bases at the 5′ end. Interestingly, this pattern does not exist in insects and plants. Further analysis revealed that this pattern might have resulted from the changes in Dicer number and miRNA sorting by Ago proteins. From plants to mammals, the number of Dicer genes decreases from more than 4 to only 1, which means that the Dicer gene in mammals has greater diversity in function. One possible reason for the Dicer gene decrease in mammals may be that mammals show different immune mechanisms from insects and plants. In both insects and plants, Dicer genes are necessary for anti-virus protection [27]. In contrast, mammals have evolved other innate immune mechanisms for anti-virus protection [28]. Therefore, the Dicer gene related with immune response is not necessary for mammals. As a result, the integration of Dicer genes makes miRNAs more diverse. Under the pressure of a complex environment, mammalian cells need functions that are more diverse. Therefore, newly evolved miRNAs tend to have a preference for the non-U base at the 5′ end, which may be helpful in switching perfect miRNA-mRNA binding to imperfect miRNA-mRNA binding. A main result of the imperfect binding is that one miRNA will have more binding sites on one gene and will have more targets. Therefore, it will be more diverse in functions.

## Materials and Methods

### Data of miRNA

We downloaded miRNA family data from miRBase (November, 2009)[29]. We calculated the conservation score for the miRNAs of human (Homo sapiens, hsa), mouse (Mus musculus, mmu), fly (Drosophila melanogaster, dme), worm (Caenorhabditis elegans, cel), Arabidopsis (Arabidopsis thaliana, ath), and rice (Oryza sativa, osa) using the method presented by Zhang et al.[4], which defined the conservation score of an miRNA using the number of its family members. The method is validated by SNP analysis [4] and evolution analysis of the miRNA transcriptional network [30].

We classified miRNAs into less-conserved and conserved based on the miRNA family data presented in miRBase. For example, for a human miRNA, if it does not have other family members (in human or other species), we take it as less-conserved, otherwise, we take it as conserved. miRNAs that belong to a miRNA family have the same seed regions but most of them do not have the same sequences at miRNA gene level. We also counted the number of family members of for each human miRNA and took this count as its conservation score. Using this score and miRNA expression data by Liang et al.[31], we observed the positive correlation of miRNA conservation and its expression level, as reported by a previous study [13]. This suggests that this score could be a useful metric to evaluate miRNA conservation. Because this metric depends on the miRNA family data, it is not perfectly accurate and will be improved when more miRNA family data becomes available in the future.

We obtained experimentally supported miRNA targets from TarBase [22] and predicted miRNA targets from TargetScan [32].

### miRNA sequences features

We obtained the sequences of MIRs and pre-miRs of the above six species from miRBase [29]. We focused on 16 sequence features, the ACGU content of pre-miRs (Features 1 to 4), the ACGU content of MIRs (Features 5 to 8), the ACGU content of non-MIRs (Features 9 to 12), and the bases at cleavage sites (Features 13 to 16). Features 1 to 12 are real numbers, and Features 13 to 16 are characters (A, C, G, or U).

### Statistical computing

All statistical analyses were performed using R, a statistical computing language (http://www.r-project.org/). We used Cluster 3.0 for clustering analysis.

## Supporting Information

Figure S1 **Distribution of the base content in four points at cleavage sites (as shown in** **Figure 2** **) for conserved miRNAs and less-conserved miRNAs in six species.** The logos are produced by WebLogo (http://weblogo.berkeley.edu/logo.cgi).
(TIF)
