# Supplementary figures and images for: Sequence Fingerprints of MicroRNA Conservation
Source: PLoS One. 2012 Oct 24;7(10):e48256. doi: 10.1371/journal.pone.0048256 (PMC3480475; doi:10.1371/journal.pone.0048256)

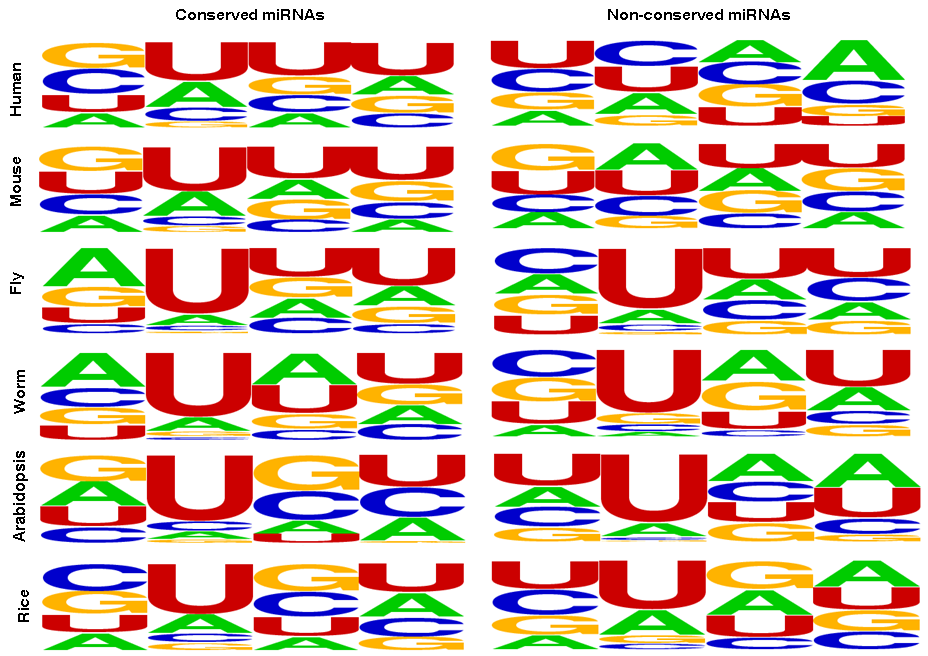

Supplement: Figure S1 — Distribution of the base content in four points at cleavage sites (as shown in Figure 2 ) for conserved miRNAs and less-conserved miRNAs in six species. The logos are produced by WebLogo (http://weblogo.berkeley.edu/logo.cgi). (TIF) [file pone.0048256.s001.tif]
